# The Effect of Genetics on Caries Risk Assessment: *MMP3* (rs679620) and *VDR* (rs731236) Gene Polymorphisms

**DOI:** 10.3290/j.ohpd.c_2755

**Published:** 2026-07-03

**Authors:** Seda Selvi, Pınar Yılmaz Atalı, Ömer Birkan Ağralı, Beste Tacal Aslan, Özlem Özge Yılmaz, Tolga Polat, Korkut Ulucan

**Affiliations:** a Seda Selvi Assistant Professor, Yeditepe University Faculty of Dentistry, Department of Restorative Dentistry, Istanbul, Turkey. Conceptualisation, methodology, formal analysis, investigation, writing – original draft preparation.; b Pınar Yılmaz Atalı Professor Dr, Marmara University Faculty of Dentistry, Department of Restorative Dentistry, Istanbul, Turkey. Conceptualisation, methodology, investigation, writing –original draft preparation, writing – review and editing.; c Ömer Birkan Ağralı Associate Professor, PhD, Marmara University Faculty of Dentistry, Department of Periodontology, Istanbul, Turkey. Methodology, investigation, writing –original draft preparation, writing –review and editing.; d Beste Tacal Aslan Assistant Professor, Marmara University Faculty of Dentistry, Department of Basic Medical Sciences, Istanbul, Turkey. Investigation, writing –original draft preparation, writing – review and editing.; e Özlem Özge Yılmaz Research Assistant, Marmara University Faculty of Dentistry, Department of Basic Medical Sciences, Istanbul, Turkey. Methodology, formal analysis, data curation.; f Tolga Polat Research Assistant, Marmara University Faculty of Dentistry, Department of Basic Medical Sciences, Istanbul, Turkey. Methodology, formal analysis, data curation.; g Korkut Ulucan Professor, Marmara University Faculty of Dentistry, Department of Basic Medical Sciences, Istanbul, Turkey. Writing – review and editing, supervision.

**Keywords:** caries risk assessment, genetics, gene polymorphisms, MMP3, VDR

## Abstract

**Purpose:**

This study evaluated the impact of gene polymorphisms in *VDR* and *MMP3* on caries and the efficacy of genetic factors in a novel caries risk model.

**Methods and Materials:**

After oral examination of male individuals (aged 20–44), a diagnosis was made according to the decayed, missing and filled teeth (DMFT) index: Group 1 ‘high caries risk’ (DMFT ≥ 14); Group 2 ‘low caries risk’ (DMFT ≤ 5) (n = 162). Participants’ detailed anamneses were recorded. Periodontal indices, saliva buffering capacity/BC, salivary quantity of *Streptococcus mutans*/SM and *Lactobacillus* spp./LB were measured. After DNA isolation, genotyping of the *MMP3* (rs679620) and *VDR* (rs731236) genes was conducted using real-time polymerase chain reaction (PCR). A stepwise regression analysis was conducted to evaluate the explanatory contributions of independent variables to DMFT variability. The results were considered significant at *p* < 0.05.

**Results:**

Significant differences were observed between the groups in terms of the plaque index, gingival index, probing depth, bleeding on probing, salivary flow rate, BC, salivary quantity of LB, frequency of consuming sugary foods/drinks, frequency of tooth brushing, and the use of preventive dental treatment (*p* < 0.001). The *MMP3 *rs679620 polymorphism significantly affected caries risk (*p* < 0.001), while the VDR polymorphism was not significant (*p* = 0.862). An explanatory regression model for caries experience, created by stepwise regression analysis, explained 61.1% of the DMFT.

**Conclusion:**

Adding genetic factors to the caries risk model may have a significant effect on the accuracy of the model.

Dental caries is a very common pathological condition that shows a significant increase in cumulative prevalence with age.^14,27^ Research has demonstrated that the financial burden associated with dental caries treatment represents a considerable proportion of global health expenditure, with the global cost of dental diseases exceeding 540 billion dollars in 2015. This not only poses a significant challenge to global health but also has substantial financial implications for individuals, communities and healthcare systems.^20,29,35^

Caries lesion formation in dental hard tissues has a multifactorial aetiology. Host (tooth), pathogenic plaque, diet and time are the primary factors affecting carious lesion formation. In addition, the use of antibacterial agents, the presence of dental sealants, saliva (flow rate, buffering capacity, pH, composition), the amount of sugar consumed, the plaque pH, the calcium‒phosphate (Ca‒PO_4_) level, education level, and socioeconomic status, etc., are also effective in caries lesion formation.^2^

Although the role of various risk factors in dental caries is questioned, individual differences may explain why some develop caries at different rates despite exposure to the same risk factors. It can thus be inferred that the observed disparity in caries susceptibility among the general population indicates the pivotal role of immunological and genetic factors in the aetiology of this condition. In consideration of these findings, numerous studies have indicated that the genetic component contributing to the variation in caries risk score is within the range of 49.1–62.7%.^13^ Caries risk may be influenced by numerous loci with minor individual contributions. Polymorphisms in genes regulating enamel formation, immunity, salivary composition/function, taste receptor mechanism and diet may play a role in caries lesion formation.^43^ The enamel formation has been demonstrated as a potential contributing factor to the susceptibility to caries.

Recent studies have identified host-derived matrix metalloproteinases (MMPs) in the oral cavity and demonstrated that they regulate mineralisation during tooth development.^5,17,42^ The presence of MMPs along the dentoenamel (DEJ) line has been demonstrated to lead to rapid progression of caries along this line.^12^ Studies have demonstrated that *MMP2* and *MMP3*, two members of the MMP family, have the capacity to accelerate the progression of caries lesions in the dentin and periapical regions.^17^ It has been hypothesised that MMP-3 may contribute to the degradation of these matrix components during the caries process. It has been proposed as a potential candidate gene for caries susceptibility and progression8.

Vitamin D plays a vital role in regulating the phosphate and calcium ion mechanisms involved in protecting and strengthening teeth.^12,15^ The most biologically active metabolite of vitamin D, 1,25(OH)_2_D_3_, exerts its effects by binding to an intracellular vitamin D receptor (VDR). The relationship between vitamin D receptor polymorphisms and the susceptibility to dental caries and periodontal health has been the subject of numerous studies.^12,33,48^

Caries risk is defined as the probability of caries lesion formation in the teeth of individuals within a certain period of time.^26,34^ The risk of caries lesion formation, which has a multifactorial aetiology, is evaluated by analysing all aetiological factors together.^2^ The procedures employed by dental professionals to control caries lesions are contingent upon the caries risk status and lesion activity of the patients in question. It is therefore crucial that dentists are able to accurately assess both the activity of caries lesions and caries risk assessment (CRA) in order to provide appropriate treatment.^36^ The American Dental Association (ADA), Caries-Risk Assessment Tool (CAT), Caries Management by Risk Assessment (CAMBRA), Cariogram, and National University of Singapore Caries Risk Assessment (NUS-CRA) are different caries risk prediction models that are widely used today. All of the aforementioned models incorporate solely environmental factors.^29,32^

In light of the studies conducted to date, the aim of this study was to investigate the effects of *VDR* and *MMP3* gene polymorphisms, which are effective in tooth mineralisation, and other caries risk factors on caries risk modelling.

## METHODS AND MATERIALS

A minimum of 151 individuals was determined to be required to achieve a power of 95% (1-β), a confidence interval of 95% (1-α), an effect size of 0.321, and a significance level of 0.05 (GPower V3.1.9.6, Düsseldorf, Germany).^33^

A total of 648 male individuals who were referred to the Marmara University Faculty of Dentistry Hospital for treatment were diagnosed according to their caries and missing and filled teeth (DMFT) index after routine intraoral examination. Individuals who met the established criteria were separated into two distinct groups. The first group consisted of individuals with high caries risk (HG) (DMFT ≥ 14, n = 79), and the second group consisted of individuals with low caries risk (LG) (DMFT < 5, n = 83) (Fig 1).

The study population comprised individuals aged between 20–44 years, male, without any genetically inherited disease in themselves or first-degree relatives, without any systemic disease and regular drug usage. Individuals who had received radiotherapy/chemotherapy, were receiving hormone therapy, had recently used vitamin D supplements, were taking medication that affected salivary flow rate, and were undergoing orthodontic treatment were excluded from the study.

The participants completed an oral health questionnaire comprising two sections. The first section pertained to demographic and socioeconomic data, while the second section addressed oral health-related behaviours.^44^

In order to guarantee uniformity in the classification process, all participants were evaluated by a faculty member and a research assistant who had completed the same undergraduate programme. After clinical and radiological evaluation, past caries experience (DMFT index) was calculated according to the World Health Organization (WHO) criteria.

The plaque index (PI), developed by Silness and Löe^38^, was employed to ascertain the quantity of supragingival microbial dental plaque (MDP). Löe and Silness gingival index (GI)^21^ was measured by running the periodontal probe along the soft tissue wall of the gingival groove. The probing depth (PD) was determined by measuring the distance between the bottom of the pocket and the free gingival margin. After the periodontal pocket was probed, (+)/(–) bleeding on probing (BOP) values were given according to the presence or absence of bleeding in the pocket.^19^ The percentage of BOP was calculated and recorded in the patient record form together with all measured parameters.

Stimulated salivary flow rate measurement was calculated in ml/min using an autoclave-sterilised measurement tube (tube with a measure), a funnel and sugar-free chewing gum. A total of 1.5 ml of saliva was collected from the participants (0.5 ml for saliva buffering capacity measurement and 1 ml for saliva *Streptococcus mutans*/SM (S. mutans) and *Lactobacillus*/LB count measurement). The collected samples were delivered to the microbiology laboratory for measurement during the day. Saliva buffer capacity was determined using the Ericsson method.^45^ The quantitative PCR method was used to determine the levels of LB and SM in the saliva sample obtained from the patient.

DNA was isolated from venous blood samples using the G-spin Total DNA Isolation Kit (Intron Biotechnology, South Korea). Genotyping of *VDR* rs731236 and *MMP3* rs679620 polymorphisms was performed via RT-PCR on a StepOnePlus device (Thermo Fisher Scientific) with TaqMan single-nucleotide polymorphism (SNP) genotyping assay kits (cat. no. 4351379) (Table 1). The protocol of the kit was applied.^28^

**Table 1:** Sequences of TaqMan probe used for genotyping *MMP3* rs679620 and *VDR* TaqI rs731236 polymorphisms

		DNA sequence (5’→3’)
*MMP3* rs679620	VIC/FAM	CTAACAAACTGTTTCACATCTTTTT[C/T]GAGGTCGTAGTAGTTTTCTAGATAT
*VDR* TaqI rs731236	VIC/FAM	TGGACAGGCGGTCCTGGATGGCCTC[A/G]ATCAGCGCGGCGTCCTGCACCCCAG


When performing the analysis on the Step One Plus Real-Time PCR System (Applied Biosystems) device, for the *MMP3* rs679620 polymorphism, the G nucleotide emits green light, and the A nucleotide emits blue light. For the *VDR* Taq1 rs731236 polymorphism, the G nucleotide glows blue, and the A nucleotide glows green.

### Statistical Evaluation

The data were analysed using the IBM SPSS Statistics software, version 23 (Chicago, USA). Independent samples t-tests were employed for the comparison of data that were normally distributed, whereas Mann–Whitney U tests were utilised for the comparison of data that were not normally distributed. In order to compare the categorical data according to the various groups, Pearson’s chi-square test, Fisher’s exact test and Yates correction were employed. Multiple comparisons were performed with the Bonferroni-corrected Z test. Binary logistic regression analysis was used to ascertain the risk factors associated with high caries risk. The analysis results are presented as the frequency (percentage) for categorical variables and as the mean ± standard deviation and median (minimum‒maximum) for quantitative variables. Genotype distributions were tested for Hardy–Weinberg equilibrium using the chi-square test. Stepwise regression analysis was applied as an exploratory variable selection method to evaluate the explanatory contributions of independent variables to DMFT variability. Regression analyses were performed for explanatory purposes rather than to develop a predictive clinical model. The level of statistical significance was set at *p* < 0.050.

## RESULTS

A statistically significant discrepancy was identified between the distributions of LB categories according to the groups (*p* = 0.001). While 48.2% of the individuals in the LG had LB concentrations less than 105 CFu/ml, 22.8% of those in the HG had LB concentrations less than 105 CFu/ml. While 51.8% of those with LB counts higher than 105 CFu/ml were in the LG, 77.2% of those with LB counts higher than 105 CFu/ml were in the HG. No statistically significant difference was found between the distributions of SM categories according to the groups *p* = 0.097) (Table 2).

**Table 2:** Comparison of the distribution of categorical variables according to low (LG) and high (HG) caries risk group

	LG	HG	Total	Test statistics	*p*
	n (%)	n (%)	n (%)		
**Salivary LB amount (CFu/ml)**					
< 105	40 (48.2)	18 (22.8)	58 (35.8)	11.368	0.001*
> 105	43 (51.8)	61 (77.2)	104 (64.2)		
**Salivary SM amount (CFu/ml)**					
< 106	25 (30.1)	14 (17.7)	39 (24.1)	2.76	0.097**
> 106	58 (69.9)	65 (82.3)	123 (75.9)		
**Frequency of dental visits**					
Every 6 months	26 (59.1)	16 (40)	42 (50)	2.339	0.126***
Once a year	18 (40.9)	24 (60)	42 (50)		
**How many times a day do you brush your teeth?**					
Every other day	3 (3.6)a	6 (7.6)a	9 (5.6)		
Once a day	12 (14.5)a	26 (32.9)b	38 (23.5)		
Twice a day	62 (74.7)a	40 (50.6)b	102 (63)	12.145	0.016*
Three times a day	5 (6)a	7 (8.9)a	12 (7.4)		
More than three times a day	1 (1.2)a	0 (0)a	1 (0.6)		
**Do you use dental floss or an interdental brush?**					
Yes	43 (51.8)	42 (53.2)	85 (52.5)	0.03	0.863*
No	40 (48.2)	37 (46.8)	77 (47.5)		
**Frequency of use of dental floss or interdental brush?**					
Every other day	18 (42.9)	19 (45.2)	37 (44)	0.758	0.685*
Once a day	21 (50)	18 (42.9)	39 (46.4)		
More than once a day	3 (7.1)	5 (11.9)	8 (9.5)		
**Do you do/have any application for preventive dental treatment?**					
Yes	9 (10.8)	7 (8.9)	16 (9.9)	0.025	0.873*
No	74 (89.2)	72 (91.1)	146 (90.1)		
**Do you often feel that your mouth is dry and feel the need to drink something liquid?**					
Yes	16 (19.3)	22 (27.9)	38 (23.5)	1.213	0.271***
No	67 (80.7)	57 (72.2)	124 (76.5)		
**Do you have difficulty chewing food and feel the need to drink fluids during eating?**					
Yes	11 (13.3)	12 (15.4)	23 (14.3)	0.026	0.872***
No	72 (86.8)	66 (84.6)	138 (85.7)		
**Do you smoke?**					
Yes	38 (45.8)	31 (39.2)	69 (42.6)	0.709	0.400*
No	45 (54.2)	48 (60.8)	93 (57.4)		
*Pearson chi-square test; **Fisher’s exact test; ***Yates Correction; a, b: There is no difference between groups with the same letter.					

There was a statistically significant difference in the frequency of brushing teeth during the day between the LG and HG (*p* = 0.016). Differences were observed between those who brushed once a day and those who brushed twice a day (Table 2).

Binary logistic regression analysis was used to analyse the risk factors affecting high caries risk. When the model was analysed multivariately, the risk of high caries increased 0.048 times as saliva buffering increased (*p* = 0.001). The risk of high caries in those with a PI of 1–2 was 126.671 times greater than that in those with a PI of 0–1 (*p* < 0.001). Those with a PI of 2–3 had a 105.553-fold greater caries risk than those with a PI of 0–1 (*p* = 0.007). The risk of high caries was 182.035 times greater in those with a BOP ≥ 10 than in those with BOP < 10 (*p *= 0.001). The risk of high caries was 31.743 times greater in those who consumed sugary food and beverages for two or more meals a day than in those who consumed fewer than one meal a day (*p* = 0.029). Other variables were not found to be risk factors for high caries risk (*p* > 0.050) (Table 3).

**Table 3:** Investigation of risk factors affecting high caries risk by logistic regression analysis

	LG	HG	Univariate		Multivariate	
	n (%)	n (%)	OR (%95 CI)	p	OR (%95 CI)	p
Age	25.98 ± 5.41	28.65 ± 8.71	1.053 (1.007–1.101)	**0.022**	0.985 (0.872–1.113)	0.808
Saliva flow rate	1.73 ± 0.64	1.31 ± 0.5	0.257 (0.135–0.491)	**< 0.001**	0.221 (0.031–1.6)	0.135
Saliva buffering	4.97 ± 0.51	4.59 ± 0.7	0.347 (0.199–0.607)	**< 0.001**	0.048 (0.008–0.287)	**0.001**
**Plaque index category**						
0–1	81 (77.9)	23 (22.1)	Reference			
1–2	1 (2.7)	36 (97.3)	126.783 (16.481 – 975.277)	**< 0.001**	126.671 (9.589 – 1673.412)	**< 0.001**
2–3	1 (4.8)	20 (95.2)	70.435 (8.968 – 553.216)	**< 0.001**	105.553 (3.607 – 3088.873)	**0.007**
**Gingival index category**						
0–1	83 (68.6)	38 (31.4)	Reference			
1–2	0 (0)	31 (100)	–	–	–	–
2–3	0 (0)	10 (100)	–	–	–	–
PD category	1.35 ± 0.29	2.18 ± 0.93	143.078 (31.228 – 655.537)	**< 0.001**	–	–
B.O.P. category						
< 10	82 (74.5)	28 (25.5)	Reference			
≥ 10	1 (1.9)	51 (98.1)	149.357 (19.713 – 1131.602)	**< 0.001**	182.035 (8.205 – 4038.688)	0.001
How many meals do you eat a day?						
Once a day	0 (0)	1 (100)	Reference			
Two or more per day	83 (51.6)	78 (48.4)	–	–	–	–
How many snacks do you eat or drink per day?						
Less than one a day	10 (45.5)	12 (54.5)	Reference			
Once a day	28 (60.9)	18 (39.1)	0.536 (0.192–1.496)	0.234	5.176 (0.305–87.692)	0.255
Two or more per day	45 (47.9)	49 (52.1)	0.907 (0.357–2.304)	0.838	6.629 (0.435–101.055)	0.174
How many meals a day do you consume sugary foods/drinks?						
Less than one a day	18 (72)	7 (28)	Reference			
Once a day	37 (50)	37 (50)	2.571 (0.96–6.884)	0.060	13.064 (0.617–276.834)	0.099
Two or more per day	28 (44.4)	35 (55.6)	3.214 (1.177 – 8.777)	**0.023**	31.743 (1.429 – 705.053)	**0.029**
Cox&Snell R^2^ = %56.8; Nagelkerke R^2^ = %75.8; Frequency (percentage).						

There was a statistically significant difference in *MMP3* distribution between the HG and LG groups (*p* < 0.001). Here, a difference was observed between those with GA and those with GG. While the percentage of GA in the LG group was 25.3%, the percentage of GA in the HG group was 72.2%. While the percentage of those with GGs in the LG group was 51.8%, the percentage of those with GGs in the HG group was 11.4%. Hardy–Weinberg equilibrium (HWE) analysis performed in the low-risk group (LG) showed that the *MMP3* genotype distribution deviated from equilibrium (*p* < 0.001). In contrast, the *VDR* genotype distribution in the LG group was consistent with the Hardy–Weinberg equilibrium (*p* = 0.500). No statistically significant difference was found between the *VDR* distributions according to the groups (*p* = 0.862) (Table 4).

**Table 4:** Comparison of *MMP3* and *VDR* distributions according to groups

	Group		Total	Test statistics	p*
	LG	HG			
*MMP3*					
AA	19 (22.9)a	13 (16.5)a	32 (19.8)	39.897	**< 0.001**
GA	21 (25.3)a	57 (72.2)b	78 (48.1)		
GG	43 (51.8)a	9 (11.4)b	52 (32.1)		
HWE *p* value	< 0.001				
*VDR*					
AA	31 (37.3)	31 (39.2)	62 (38.3)	0.296	0.862
AG	37 (44.6)	32 (40.5)	69 (42.6)		
GG	15 (18.1)	16 (20.3)	31 (19.1)		
HWE *p* value	0.500				
Allele count for *MMP3*					
A allele count	59 (35.5)	83 (52.5)	142 (43.8)	9.491	**0.002**
G allele count	107 (64.5)	75 (47.5)	182 (56.2)		
Allele count for *VDR*					
A allele count	99 (59.6)	94 (59.5)	193 (59.6)	0.001	0.979
G allele count	67 (40.4)	64 (40.5)	131 (40.4)		
* Pearson chi-square test; a, b: There is no difference between groups with the same letter; Frequency (percentage). * Hardy–Weinberg equilibrium (HWE) was assessed using the chi-square test in the control (LG) group.					

A statistically significant difference was identified between the distributions of allele numbers for the *MMP3* gene according to the groups (*p* = 0.002). The A allele rate was 35.5% in the LG group and 52.5% in the HG group. The G allele rate was 64.5% in the LG group and 47.5% in the HG group. No statistically significant difference was found between the *VDR* allele number distributions according to the groups (*P* = 0.979) (Table 4).

In the odds ratio analysis of the *MMP3* polymorphism, individuals with the GA genotype had a significantly higher risk of being in the high-caries group compared to those with the AA genotype (OR = 3.97 [1.63–9.68], *p *= 0.002). In contrast, the GG genotype showed a protective effect (OR = 0.31 [0.12–0.78], *P* = 0.013). In the recessive model (GG vs GA+AA), a strong protective association was observed (OR = 0.12 [0.05–0.32], *p *< 0.001), whereas the dominant model did not show a statistically significant association (*p* = 0.327). Allelic analysis revealed that the A allele was associated with an increased caries risk (OR = 2.01 [1.29–3.12], *p *= 0.002). No statistically significant association was found between *VDR* polymorphism and caries risk in any genetic model (*p *> 0.05) (Table 5).

**Table 5:** Genotype comparisons of *MMP3* and *VDR* polymorphisms according to caries risk groups (HG vs LG)

Genotype comparison	HG vs LG group OR (95% confidence interval)	
	OR (95% confidence interval)	*p* value
*MMP3* rs679620		
GA vs AA	3.97 (1.63–9.68)	**0.002**
GG vs AA	0.31 (0.12–0.78)	**0.013**
GG vs GA+AA (recessive model)	0.12 (0.05–0.32)	**< 0.001**
GG+GA vs AA (dominant model)	1.51 (0.66–3.47)	0.327
Allelic (A vs G)	2.01 (1.29–3.12)	**0.002**
*VDR* rs731236		
AG vs AA	0.87 (0.45–1.68)	0.675
GG vs AA	1.06 (0.47–2.38)	0.884
GG vs GA+AA (recessive model)	1.14 (0.55–2.36)	0.726
GG+AG vs AA (dominant model)	0.92 (0.49–1.71)	0.799
Allelic (A vs G)	1.00 (0.66–1.51)	0.979
IndicatesP < 0.05.		

Stepwise regression analysis was performed to analyse the variables affecting DMFT. The model was analysed in eight steps, and the models were found to be statistically significant (*p* < 0.001). Model 1 explained 45% of the DMFT, Model 2 explained 55.6%, Model 3 explained 56.3%, Model 4 explained 56.8%, Model 5 explained 58.4%, Model 6 explained 60.4%, Model 7 explained 60.9%, and Model 8 explained 61.1%. When the *MMP3* variable was added to Model 1, the percentage of explanation in R2 changed by 10.6%; when the *VDR* was added to Model 7, the percentage of explanation change was 2% (Table 6).

**Table 6:** Changes in the factors affecting DMFT and examination of the significance of the models

Model	R2	Corrected R2	Change Statistics			Model Significance	
			R2 change	F change	*p*	F	*p*
1 plaque	0.671^a^	0.450	0.450	130.79	**< 0.001**	130.79	**< 0.001**
2 *MMP3*	0.745^b^	0.556	0.106	18.845	**< 0.001**	65.885	**< 0.001**
3 BOP	0.750^c^	0.563	0.007	2.473	0.118	50.493	**< 0.001**
4 LB	0.754^d^	0.568	0.005	1.956	0.164	41.032	**< 0.001**
5 sugary food/drinks	0.764^e^	0.584	0.016	3.021	0.052	30.931	**< 0.001**
6 buffer	0.777^f^	0.604	0.019	7.528	**0.007**	29.153	**< 0.001**
7 brushing	0.780 ^g^	0.609	0.005	0.483	0.748	19.334	**< 0.001**
8 *VDR*	0.782^h^	0.611	0.002	0.357	0.701	16.479	**< 0.001**
^a^ Predictors: (Constant), Plaque Index. ^b^ Predictors: (Constant), Plaque Index, *MMP3* (GA), *MMP3* (GG). ^c^ Predictors: (Constant), Plaque Index, *MMP3* (GA), *MMP3* (GG), BOP. ^d^ Predictors: (Constant), Plaque Index, *MMP3* (GA), *MMP3* (GG), BOP, LB category (Y). ^e^ Predictors: (Constant), Plaque Index, *MMP3* (GA), *MMP3* (GG), BOP, LB category (Y), How many meals a day do you consume sugary foods/drinks? (two or more per day), How many meals a day do you consume sugary foods/drinks? (once a day). ^f^ Predictors: (Constant), Plaque Index, *MMP3* (GA), *MMP3* (GG), BOP, LB category (Y), How many meals a day do you consume sugary foods/drinks? (two or more per day), How many meals a day do you consume sugary foods/drinks? (once a day), saliva buffering. ^g^ Predictors: (Constant), Plaque Index, *MMP3* (GA), *MMP3* (GG), BOP, LB category (Y), How many meals a day do you consume sugary foods/drinks? (two or more per day), How many meals a day do you consume sugary foods/drinks? (once a day), Saliva buffering, frequency of tooth brushing (once a day), frequency of tooth brushing (twice a day), frequency of tooth brushing (three times a day). ^h^ Predictors: (Constant), Plaque Index, MMP3 (GA), MMP3 (GG), B.O.P., LB category (Y), How many meals a day do you consume sugary foods/drinks? (two or more per day), How many meals a day do you consume sugary foods/drinks? (once a day), saliva buffering, frequency of tooth brushing (once a day), frequency of tooth brushing (twice a day), frequency of tooth brushing (three times a day), *VDR* (AG), *VDR* (GG). ^ı^ Dependent variable: Dmft.							

## DISCUSSION

We conducted this study to investigate the importance of *VDR* and *MMP3* SNPs in caries formation and to analyse gene‒environmental influences on caries risk. It was hypothesised that individuals with more caries lesions would be genetically susceptible to caries. In most studies evaluating caries risk, caries risk groups were defined according to the DMFT index, since past caries experience is an important risk indicator for future caries development.^41^ In our study, based on the World Health Organization DMFT classification data,^46^ the study subjects were divided into two extreme DMFT groups: DMFT ≥ 14 and DMFT < 5, to reduce heterogeneity, as in similar genetic studies.^47^

According to the criteria of the WHO, those aged 15–24 years were classified as young adults, those aged 25–44 years were classified as adults, and those aged 45–64 years were classified as middle-aged adults.^31^ Compared with those of young adults, changes in the salivary flow rate due to systemic diseases and regular medication use, increases in the amount of dental plaque, restorations without proper contour‒contact relationships, exposed root surfaces and the presence of secondary caries are more common in adults.^34^

Women are more prone to caries than men due to factors such as differences in saliva flow rate and composition, dietary habits, genetic variations, and hormonal fluctuations during pregnancy.^11^ Moreover, females tend to have fewer extracted teeth than males, likely due to better oral hygiene.^23,34^ Studies have shown that, during childhood and adolescence, girls exhibit higher DMF scores compared to boys.^34,40^ Hormones, particularly oestrogen, are known to elevate *MMP2* levels, with some research suggesting that oestrogen influences *MMP3* expression.^24^ While certain studies show a correlation between oestrogen deficiency and increased *MMP3*, others report no significant oestrogen effect. Furthermore, a link has been found between *MMP3* G allele carriers and dental caries resistance in females. These findings indicate that sex and the menstrual cycle can impact gene expression.^17^ Additionally, some studies have identified X-linked loci related to both caries susceptibility and protection.^22^ Given these factors, our study focused on young adult males aged 20–44, marking the first study to include only male participants.

In our study, blood samples were selected for DNA isolation, which is the first step of molecular diagnostic procedures, due to the low risk of contamination and the ease of obtaining more concentrated DNA.^1,9^

MMP-3, also known as stromelysin, is an important matrix metalloproteinase involved in the degradation of the extracellular matrix in physiological and pathological processes. The rs679620 variant in the *MMP3* gene is a missense polymorphism that causes a lysine-to-glutamine amino acid substitution and may affect gene function. Silva et al reported a significant association between this variant and deep caries lesions and periapical disease, suggesting that rs679620 may be a genetic marker for lesion progression in dentine and periapical pathologies.^25^ The studies conducted by Borilova Linhartova et al^6^ on children in the Czech population and Karayasheva et al^17^ on adults in the Bulgarian population found no evidence of a relationship between the *MMP3* rs679620 region and the development of dental caries. In our study, a statistically significant difference was found between the groups in terms of *MMP3* distribution (*p* < 0.001). In addition, Hardy–Weinberg equilibrium (HWE) analysis performed in the control group (LG) revealed a deviation from equilibrium for the *MMP3* genotype distribution. This deviation may be attributed to factors such as sampling variability, population stratification, or the relatively limited sample size, and therefore should be interpreted with caution. The different results obtained in these studies may be attributed to the fact that the studies were carried out on different ethnic origins and different age groups. Studies in the current literature demonstrating the direct effect of this polymorphism on MMP-3 expression or enzymatic activity, or validating its functional role in oral tissues, are limited. Therefore, the association identified in our study should be interpreted as an indicator of genetic predisposition rather than direct biological causality, and further molecular studies are required to clarify the functional consequences of rs679620 in the oral environment.

There was no statistically significant difference in the *VDR* distributions among the groups in our study (*p* = 0.862). When we examined studies on *VDR* polymorphisms in the last 20 years in the literature, we reviewed seven studies on the rs731236 region. Izakovicova et al,^16^ Yuan-yuan et al,^18^ Xiurong et al,^33^ and Yu et al^48^ reported that the *VDR* rs731236 gene polymorphism was not associated with caries in parallel with our study. In a study conducted by Cogulu et al^9^ among children aged 6–12 years in the Turkish population, Hu et al^14^ among adults aged 30–67 years in the Chinese population, and Aribam et al^3^ among children aged 6–12 years in an Indian population, the *VDR* rs731236 gene polymorphism was reported to be associated with caries formation. This may be attributed to the fact that although Cogulu et al conducted their study on individuals of Turkish ethnicity, the study group consisted of the child population, Hu et al conducted their study on individuals of Chinese ethnicity, and Aribam et al conducted their study on a child population of Indian ethnicity.

In our study, the odds ratio analysis further supports the role of the *MMP3* rs679620 polymorphism in caries susceptibility. Particularly, the increased risk associated with the GA genotype and the protective effect of the GG genotype suggest a genotype-dependent influence on caries development. In contrast, the *VDR* rs731236 polymorphism did not demonstrate any significant effect, which is consistent with our primary analysis.

When the studies conducted to develop caries risk models for adults were analysed, it was found that most of the studies included only environmental risk factors. In these studies, in which different environmental factors were evaluated together, the explanatory values of the developed risk models varied between 16.6% and 77%.^7,10,37^

When we examined the results of the environmental risk factors in our study, the salivary LB amount (CFu/ml) and daily tooth brushing frequency categories differed significantly between the groups according to the distribution of categorical variables. According to the univariate evaluation of the risk factors affecting high caries risk via logistic regression analysis, age, salivary flow rate, salivary buffering capacity, PI, PD category, BOP, and frequency of daily consumption of sugary food/drink categories were significantly different between the groups.

While univariate evaluation reveals only the effect of the investigated factor on the groups, multivariate evaluation reveals the effect of the factor on the groups by taking other variables into account. For this reason, we included the factors that created significant differences according to the results of multivariate evaluation and the factors that created significant differences as a result of the distribution of categorical variables in the explanatory regression model for caries experience we created.

Considering the caries risk models that include both environmental and genetic factors, the accuracy of the caries risk model in the study conducted by Slayton et al in 2005^39^ was 26.8%, the accuracy of the model that best explained the past caries experience in the study conducted by Patir et al^30^ was 40.2%, the accuracy of the model created by Yıldız et al^47^ was 87.8%, and Pang et al^29^ determined the area under the curve (AUC) of the model as 0.73 (which indicates high discrimination ability) in their study via a machine learning algorithm.

In our study, regression analyses were performed to evaluate factors associated with caries formation. An explanatory model including PI, BOP, frequency of sugary snack consumption, salivary buffering capacity, tooth brushing frequency, salivary lactobacilli count, and the *MMP3* rs679620 and *VDR* rs731236 polymorphisms was constructed to assess their combined contribution to DMFT variability. Within the study sample, these variables collectively explained 61.1% of the variance in DMFT values.

The inclusion of the PI, BOP, frequency of consumption of sugary snacks, salivary buffering, salivary lactobacilli count, and *MMP3* rs679620 and *VDR* rs731236 gene polymorphism variables in the caries risk model to be created may be sufficient to identify individuals at high and low risk.

The PI was also included in the first step of the study by Yıldız et al. Although the inclusion of the PI in the model explained 45% of the DMFTs in our study, the 77.6% prevalence in the study by Yıldız et al is thought to be due to the narrower age range of the individuals included in our study. Yıldız et al reported that salivary buffering capacity resulted in a 0.5% change in the model, whereas it resulted in a 2% change in our study. While the *DEFB1* gene increased the model percentage by 5.4%, the *TAS2R38* gene increased by 0.8%, and the *CA6* gene increased by 0.5% in the model in the study by Yıldız et al. The *MMP3* gene, which we examined in our study, caused an increase of 10.6% in our model alone.

In some of the studies in the literature, the participants were divided into different groups according to their ethnic origins, and the activities of polymorphisms of different ethnic origins were determined. In our study, individuals living in Istanbul were included, and this distinction was ignored because there were no questions about the ethnic origin of the patients in the questionnaire.

In a limited number of studies in the literature, study groups were formed on the basis of the International Caries Detection and Assessment System (ICDAS) classification, assuming that it may produce different results as a more detailed lesion severity scale, instead of the DMFT in the differentiation of risk groups.^4^ However, since these studies obtained results similar to those reported in previous studies evaluating genetic polymorphisms via the WHO criteria, we used the DMFT criteria for ease of application to differentiate our study groups.

To determine which of the different risk groups individuals are at risk, it is necessary to determine whether new caries lesions have formed by clinically following them for the last two years. In this context, the fact that the clinical follow-up step was not performed in determining the groups of individuals who participated in the study is one of the factors limiting our study. In addition, the amount of data can be increased by increasing the number of people included in our study.

Another limiting factor is that only male individuals were included in our study. Further studies conducted in this field, with a focus on female subjects, will contribute to the advancement of knowledge in this area by providing additional insights and comparisons with the findings of the current study.

Although participants were divided into extreme DMFT groups to reduce heterogeneity, this sampling strategy may limit the generalisability of the findings. The use of extreme phenotype groups can increase observed effect sizes and the proportion of explained variance; therefore, the reported R^2^ value should be interpreted within the context of the study sample rather than as representative of the general population. Furthermore, the regression analyses were performed to explore associations between clinical, behavioural, microbiological, and genetic factors and caries experience rather than to establish a predictive clinical model. Consequently, validation analyses and predictive performance measures were not included. Future population-based studies, including broader DMFT distributions and independent samples, are needed to confirm these findings.

Our study differed from previous studies investigating the effects of gene polymorphisms and environmental factors on caries risk by including only male subjects and measuring the gingival index, BOP, PD in addition to the PI. This is the first study to examine the role of *MMP3* and *VDR* gene polymorphisms in an explanatory regression model for caries experience, taking into account gene‒environment interactions, in Turkish adults.

## CONCLUSION

In conclusion, the *MMP3* rs679620 polymorphism is effective in caries formation and adding this factor to the caries risk model may lead to a significant increase in the accuracy of the model. Due to the limitations of the study, it is necessary to increase the sample size and conduct new studies to obtain more convincing and realistic analyses.

By identifying all environmental and genetic risk factors, it will be possible to accurately, easily and rapidly identify individuals with caries susceptibility in the population; thus, it will be possible to make preventive application plans to prevent caries formation in the early period. As a result of future studies, if the degree of effectiveness of the role of genetic factors in caries formation is high, it will be possible to develop genetically based treatments for the prevention of dental caries.

### Acknowledgement

The authors would like to thank Assoc. Prof. Dr. Mehmet Burak Aksu (Department of Microbiology, Faculty of Medicine, Marmara University) for his support with the microbiological analyses.

#### Statement of ethics

This study was approved by the Ethics Committee (protocol number 09.2021.292) of the Faculty of Medicine of Marmara University and was conducted in accordance with the Helsinki Declaration.

#### Consent for publication

Informed consent forms were signed by each participant included in the study.

#### Conflict of interest statement

The authors have no conflicts of interest to declare.

#### Funding sources

This study was not supported by any sponsor or funder.

#### Data availability statement

The data presented in this study are available upon request from the corresponding author.

**Fig1:** Study protocol.


